# Inhibition of Pore-Forming Proteins

**DOI:** 10.3390/toxins11090545

**Published:** 2019-09-19

**Authors:** Neža Omersa, Marjetka Podobnik, Gregor Anderluh

**Affiliations:** Department of Molecular Biology and Nanobiotechnology, National Institute of Chemistry, Hajdrihova 19, 1000 Ljubljana, Slovenia; neza.omersa@ki.si (N.O.); marjetka.podobnik@ki.si (M.P.)

**Keywords:** pore-forming proteins, pore-forming toxins, anthrax toxin, lipid membranes, pore formation, inhibitor

## Abstract

Perforation of cellular membranes by pore-forming proteins can affect cell physiology, tissue integrity, or immune response. Since many pore-forming proteins are toxins or highly potent virulence factors, they represent an attractive target for the development of molecules that neutralize their actions with high efficacy. There has been an assortment of inhibitors developed to specifically obstruct the activity of pore-forming proteins, in addition to vaccination and antibiotics that serve as a plausible treatment for the majority of diseases caused by bacterial infections. Here we review a wide range of potential inhibitors that can specifically and effectively block the activity of pore-forming proteins, from small molecules to more specific macromolecular systems, such as synthetic nanoparticles, antibodies, antibody mimetics, polyvalent inhibitors, and dominant negative mutants. We discuss their mechanism of inhibition, as well as advantages and disadvantages.

## 1. Introduction to Toxic Pore-Forming Proteins

### 1.1. Different Modes of Creating a Pore in Cellular Membranes

Plasma as well as organelle membranes are vital for cells. They protect cells from the environment, including invading organisms, enable exchange of substances either between cells and their surroundings or between different cellular compartments, cell adhesion, transport, metabolism, and flow of information via cell signaling. Thus, interfering with the integrity of membranes can disturb cellular processes and can, in extreme cases, be detrimental. During evolution, organisms from all kingdoms of life have evolved mechanisms to form pores in membranes, in order to attack other organisms or defend against them, to digest their prey, or as a part of the immune system to remove unwanted cells. Excellent reviews are available describing diverse modes of transmembrane pore formation by proteins [1,2,3,4,5,6,7,8].

**Figure 1:**
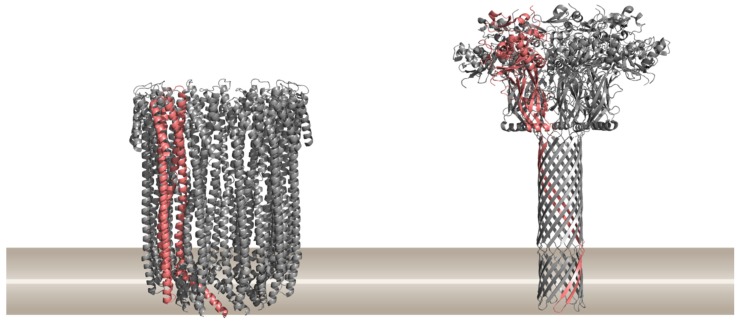
Two major classes of pore-forming proteins (PFPs) based on the structural element present in the final pore, α-helical PFPs exemplified by the cytolysin A from *Escherichia coli* (PDB ID 2WCD) on the left, and β-barrel PFPs exemplified by the anthrax toxin protective antigen pore from *Bacillus anthracis* (PDB ID 3J9C) on the right. Ribbon representations of proteins are drawn by using PyMOL [39]. A single protomer in the pore is shown in pink. The approximate position of the lipid membrane is shown in brown.

Pore-forming proteins (PFPs) are generally secreted by cells as soluble monomers that assemble into structured oligomeric complexes at the target membrane surface. Upon binding to the lipid membrane, monomers oligomerize on its surface to form structured assemblies called prepores and undergo conformational changes in order to expose hydrophobic surfaces, leading to spontaneous insertion into the lipid bilayer, pore formation, and membrane permeabilization. Pores made by PFPs are largely diverse in inner diameter, ranging from 0.7 nm as in the case of colicins [9] to the largest known pores of cholesterol-dependent cytolysins (CDCs) with diameters of 25–40 nm [8]. Depending on the size of the pore, different substances pass through, such as ions (e.g., Ca^2+^, K^+^), small molecules (e.g., adenosine triphosphate (ATP)), or large molecules (e.g., proteins) [10]. Structurally, PFPs are divided into two major classes based on secondary structure elements that frame the transmembrane channel of their pores either with α-helices (i.e., α-PFPs) [11] or β-barrels (β-PFPs) [1,2,12] (Figure 1). Three dimensional structures of soluble monomeric PFPs, prepores and pores from different families have been known to date [13,14,15,16,17,18,19,20,21,22] and several excellent reviews describe their features [10,23,24]. These structural models provide a valuable insight into the mechanism of action by PFPs and crucially contribute to a rational design of their potential inhibitors. The shapes of pores are quite diverse. Some PFPs form matrix-type toroidal pores, where the transmembrane protein units are interspersed by lipids [3], such as actinoporins [25,26], colicins [27], and proteins from the Bcl-2 family of apoptotic proteins [28]. In contrast to toroidal formations, pore walls can also be completely built of proteins, forming either compact α-barrels as in the case of α-PFP cytolysin A from *Escherichia coli* (Figure 1), or β-barrels, formed by β-PFPs such as α- and γ-toxin from *Staphylococcus aureus*, membrane attack complex/perforin (MACPF)/cholesterol-dependent cytolysins (CDCs) protein superfamily, or aerolysin-like proteins [2]. β-barrels are also formed by bacterial secretion systems of type III and IV [1,29] and binary AB type toxins, where the B component is pore-forming and allows translocation of A subunits that possess catalytic activity (e.g., diphtheria; anthrax; α-, ε-, ι-, and C2 toxins) [14,30,31,32] (Figure 1). In the case of β-barrel proteins such as MACPF/CDCs, arc-type toroidal pores partly lined with lipids as well as multimeric pores can also be built in addition to fully proteinaceous ring-shaped pores [3]. The majority of work regarding PFP inhibitors has been done on the B component (protective antigen or PA) of the anthrax toxin from *Bacillus anthracis*, hence a short description of this toxin follows. For an in-depth understanding of individual PFPs and their characteristics, an interested reader can choose from recent reviews about that topic [1,2,3,7,8,33,34,35,36,37,38].

Anthrax is a deadly disease and is considered a biological threat due to the antecedent weaponization of this agent. The component B of an anthrax toxin is responsible for the cell surface binding, whereas the component A is enzymatically active [40]. The component B is known as protective antigen (PA), while there are two distinct A components, a lethal factor (LF) and an edema factor (EF). Association between PA an LF forms the lethal toxin (LT), and interaction of PA with EF the edema toxin (ET) [41]. The pore is formed by a precursor PA_83_ binding to cell surface receptors [42,43], followed by proteolytic cleavage of PA_83_ by the protease furin, resulting in PA_63_, which oligomerizes and forms a homo-heptameric [15,16] and/or homo-octameric [44] PA prepore, which undergoes conformational changes to insert in the membrane and form a functional pore. The pore allows binding and transportation of LF or EF to the cytosol [45]. Vaccines against anthrax are available [46], but despite its poor prognosis, a widespread public immunization is unlikely due to its low incidence [47]. Consequently, searching for new strategies to protect against this disease is therefore warranted [48].

### 1.2. Effects of PFPs on Target Cells and Their Biological Roles

The best characterized and the largest group of PFPs are bacterial PFPs [10], many of which are the key virulence factors of deadly diseases and are also referred to as pore-forming toxins (PFTs). They can act on host cell physiology, tissue integrity, and immune response and cause inflammation that may interfere with antimicrobial treatment [49,50]. PFPs produced by a particular bacterium can form pores in the membrane of other bacteria, plants, animals, or humans, thereby causing disruption of membrane integrity and ion imbalance [29]. To kill other bacteria, some bacteria produce proteins such as colicins [51,52]. To attack eukaryotic cells, some bacteria express CDCs, hemolysins, and aerolysin-like proteins to promote colonization, spread, and survival within the hostile environment of a host organism [29]. In addition to bacteria, PFPs with (potential) toxic function are excreted also by eukaryotic organisms such as fungi, parasites, cnidarians, arachnids, earthworms, or plants, for the purposes of feeding or to defend against their predators. PFPs that are used in defense are also produced by vertebrates, for instance the complement membrane attack complex (MAC) to kill bacteria [17,53], or perforin to kill malignant or virus-infected cells [54], as well as proteins of the Bcl-2 family that cause apoptosis (e.g., Bak and Bax proteins) [55,56].

In this review, we describe various ways of preventing pore formation, especially of toxic PFPs. For majority of toxic PFPs, there are no effective antidotes or antitoxins developed and approved for human use [57]. These different ways and means of inhibition of PFPs can on one side help in studies of the pore-forming mechanism at the molecular level, as well as in the design of novel agents and innovative strategies for therapeutic, diagnostic, labeling, or biosensing purposes.

## 2. Modes of Preventing Pore Formation

**Figure 2:**
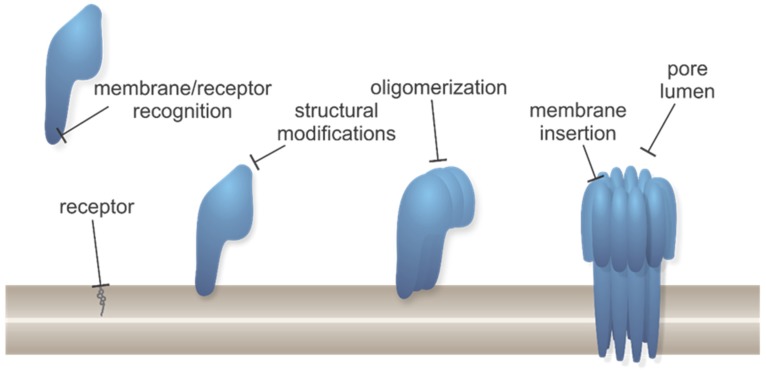
Generalized pore formation process by different types of PFPs with marked positions for possible inhibitors interfering. Protein monomers are shown in blue, lipid membrane is shown in brown, receptor for PFP binding (which can be either a specific lipid as shown here, or protein, etc.) is shown in gray.

Although structural features and properties of pores formed by PFPs are substantially diverse, their activity can be targeted in a similar manner, as the molecular mechanism of action basically follows a common pathway. Generalized steps in the molecular mechanism of pore formation of PFPs together with steps allowing potential inhibition are illustrated in Figure 2.

A range of various molecules has been developed that neutralizes the activity of toxins, the majority of them aiming for therapeutic potential [58,59]. Possible ways to inhibit the virulent effects of PFPs are by interfering either with their expression (inhibition of transcription regulators [60], protein synthesis, quorum sensing [61]), or interaction with a cognate receptor [62,63,64,65], structural modifications of membrane-bound precursors, oligomerization, membrane insertion, or pore lumen and, consequently, with the transport of molecules or ions through formed pores [66] (Figure 2). Additionally, PFP function can be also targeted indirectly by counteracting the effects of PFPs [58], such as by membrane repair [67,68,69], enhancement of blebbing and microvesicle shedding [70], and by specifically boosting or pre-activating host defense that neutralizes PFPs [38].

Antibiotics in conjunction with vaccination are set as the first line of treatment for some diseases caused by toxic PFPs (anthrax, pneumonia, etc.). Vaccination has some drawbacks, such as inconsistent efficacy, economical impracticality, or unavailability. Furthermore, the antibiotic treatment can provoke several side-effects [71], it must be given early when symptoms are nonspecific, the interval between initial exposure and the onset of treatment can be lengthy [72]. Moreover, there is a growing number of multidrug resistant bacteria secreting highly potent exotoxins with no antitoxins currently available on the market [73,74]. Therefore, development of new inhibitor scaffolds, virulence-targeted antimicrobial prophylactics, and therapeutics with a narrower spectrum, and combination therapies are needed to find more efficient treatments of increasingly resistant bacteria [75,76,77]. Here we review possible approaches for direct inhibition of pore formation by intervening with various steps of pore formation process, as outlined in Figure 2. Those strategies are worth pursuing as they offer several advantages compared with targeting the bacteria themselves: (i) Organisms are less likely to develop resistance to such scaffolds and normal microbiota remain undisturbed [73,78], (ii) mechanism of action of PFPs is well defined and is composed of distinct steps, which allow specific and targeted activity of inhibitors and consequently reduced probability of side-effects, (iii) broad application against many bacterial infections, and (iv) acting directly on a particular virulence factor to prevent as well as cure the disease [38,57,66,79,80,81]. However, it has to be noted that since alternative approaches for inhibition of PFPs have not been as widely used as antibiotics and vaccines, side-effects have not been recognized to a similar extent yet.

**Figure 3:**
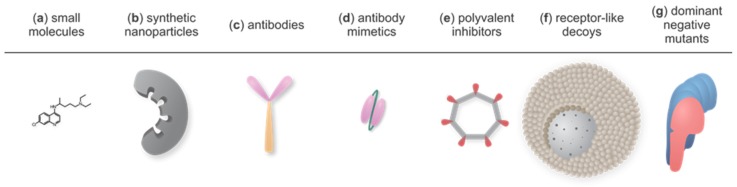
Overview of various strategies to inhibit PFPs: (**a**) Small molecules. Chloroquine as a representative molecule. (**b**) Synthetic nanoparticles. A mold with pockets for PFP binding. (**c**) Antibodies (Abs), a fragment crystallizable (Fc) region shown in orange and fragment antigen-binding (Fab) regions in pink. (**d**) Antibody mimetics. Smaller protein molecules derived from antibodies that overcome some of their weaknesses. scFv (single chain fragment variable), a fusion protein of interconnected variable regions of Fab as a representative molecule. (**e**) Polyvalent inhibitors. Cyclic scaffold (gray) with PFP-binding moieties (red) that positionally match with monomeric protein units in a pore. (**f**) Receptor-like decoys. Polymeric core enclosed by lipid bilayer containing PFP receptors. (**g**) Dominant negative mutants. Mutant protein monomer (pink) forms oligomers with the wild-type protein (blue), but such complexes fail to form pores. For clarity, individual schemes are not drawn in proportion.

Below we describe possible approaches, from small organic molecules to relatively large organic particles, peptides, or proteins and discuss their relevance. However, the standard approach used in drug design, of targeting proteins with small molecules, is not commonly employed in the case of PFPs. PFPs have a specific mode of action that involves large protein surfaces and only rarely provides cavities or binding sites that could be used for small-molecule drug development. For a successful blockage, an important role is played by the size, conformation, symmetry, and structural plasticity of the inhibitor. Therefore, the diversity of potential inhibitors encompasses small organic molecules to relatively large organic particles, peptides, or proteins. Lately, there has been an ascent of alternative binding scaffolds with similar binding characteristics as antibodies, yet overcoming some of their weaknesses, such as high cost, challenging production protocols, and low production yield [82]. Furthermore, there are some less conventional ways for the neutralization of toxin activity, for instance the utilization of dominant negative mutants [83] and receptor decoys [84,85,86]. The representatives of all discussed inhibition strategies are presented in Figure 3.

### 2.1. Small Molecules

The discovery and development of small-molecule antitoxins represent a high-priority task in modern drug design and medicinal chemistry [57,73,87], mostly because of their small size, excellent tissue penetration, long room-temperature shelf life, ease of analogue design and preparation of high-purity molecules in large quantities. Although this is an attractive research avenue, the number of studies of small-molecule PFPs inhibitors is very limited. Assorted trials are described in the review by Nestorovich and Bezrukov, 2012 [57].

Inhibition of a completely formed pore of oligomerized PFP units by small molecules is usually non-specific, meaning that the small molecule sterically hinders ion fluxes through the pore either by electrostatic or hydrophobic interactions in the lumen of the channel without binding to a specific binding site on the protein [88]. Such inhibitors can probably block only PFPs with a small pore diameter and not larger ones, e.g., CDCs. The most recognized small molecules acting that way are various chloride channel blockers. For example, they inhibit VacA, a vacuolating pore-forming binary AB type cytotoxin produced by the human pathogen *Helicobacter pylori*, as well as a variety of other non-homologous anion-selective channels [88]. Other binary toxins such as C2II component of C2 toxin, Ib of ι-toxin, and PA_63_ of anthrax toxin, can be efficiently blocked in vitro and in vivo by a drug chloroquine (Figure 3a) or its analogues with the same backbone architecture and the side-chain diversity, containing at least one positively charged quaternary ammonium group [89,90,91,92,93]. Chloroquine and other quinoline derivatives have been used in the treatment of malaria. Besides PFPs, they can also block endogenous chloride channels [94], as well as nicotinic acetylcholine receptors (nAChRs) [95] and have several recognized side-effects, such as sensorineural hearing loss, tinnitus, and vertigo [96].

Inhibition of a toxic PFP activity can also be achieved by targeting receptors and specific sites or “hot spots” involved in protein–protein or protein–lipid interactions. Small-molecule inhibitors can also occupy receptors and therefore attenuate the possibility of PFP binding. PFP receptors can be involved in diseases (e.g., cancer, Alzheimer’s disease, cystic fibrosis, or auto-immune diseases) [65,97,98,99,100] and small-molecule therapeutics, used to cure these diseases, can help develop blockers for PFP receptors [101]. In another case, inhibitors of ATP-gated purinergic receptors (P2XR) were found to inhibit *S. aureus* α-toxin membrane binding and oligomerization [102]. Examples of small-molecule inhibitors that bind PFP monomers and attenuate the binding to receptors are calixarenes (p-sulfonato-calix[n]arenes), inhibiting leukotoxins [49]. Moreover, small molecules can be also targeted to hot spots on the PFP surface responsible for oligomerization. Several natural compounds have been found that prevent different steps in pore-forming process of *S. aureus* α-toxin [103,104,105], *Streptococcus pneumoniae* pneumolysin [106,107], *Streptococcus pyogenes* streptolysin O [108], and *Listeria monocytogenes* listeriolysin O [109]. Recently, necrosulfonamide, a small-molecule inhibitor of pyroptotic PFP gasdermin D was identified, which disables protein dimers to oligomerize and form pores [110]. Cisplatin, which is one of the most effective chemotherapeutic anticancer agents, can inhibit proper heptamer assembly of anthrax toxin PA component in a noncovalent reversible manner, preventing toxicity of both factors, LF and EF [66,111]. Additionally, hexa-D-arginine can be used as a blocker of PA proteolytic cleavage and oligomerization [112]. Another strategy for inhibition is to impair membrane insertion once the prepore is already formed. For example, this can be achieved by amiodarone and bepridil, which have been used to treat cardiac arrhythmia or angina. These drugs interfere with the insertion of the PA heptamer into the endosomal membrane via neutralization of the endosomal pH, thereby blocking toxin entry into the cytosol. Those drugs, however, can have severe side-effects in high doses. For efficient PFP inhibition and reduced risk of side-effects Sanchez et al., 2007, propose a combination of different drugs at lower concentrations [113]. Toxic PFP action can also be diminished by inhibition of a non-pore-forming component of the toxin, such as numerous examples of small-molecule inhibitors of anthrax toxin component LF [18,81,114,115,116,117,118].

Perforin is one of the most important proteins in the immune system of vertebrates. It belongs to the MACPF/CDC superfamily and is able to form pores in target cells. During the process of elimination of cancer or virus-infected cells, perforin is released into the immunological synapse by cytotoxic T lymphocytes and natural killer cells. It forms transmembrane β-barrel pores on target cells and enables the passage of apoptotic proteins, which leads to cell death [19]. Specific inhibitors of perforin were identified by using high-throughput screens, particularly dihydrofuro [3,4-c]pyridinones [119], 1-amino-2,4-dicyanopyrido[1,2-a]benzimidazoles [120], and aryl-substituted isobenzofuran-1(3H)-ones [121,122]. Some of the substances were shown to have a half maximal inhibitory concentration (IC50) in the micromolar range for lysis of Jurkat cells and showed considerable inhibitory potency in the killings of target cells in cell-based cytotoxic assays. However, there has been a limited success in further development of potent inhibitors due to toxicity of compounds to the cells, poor solubility, or loss of activity in the presence of serum.

### 2.2. Synthetic Nanoparticles

Synthetic polymer nanoparticles (NPs) or “plastic antidotes” (Figure 3b) are synthetic scaffolds with affinity for target biomacromolecules and can be thus used as a tool for the inhibition of PFPs. They are synthesized by precipitation polymerization of different acrylamide monomers in the presence of a PFP [123]. Based on their architecture, they can interact with target molecules through multiple sites, however, they retain small size and ability to diffuse to many locations throughout the body. In comparison to larger bulk materials, nano-sized materials have larger surface areas and thus possess a substantial adsorbing capacity [124,125]. Hoshino et al. demonstrated that imprinted nanoparticles, which are custom-made plastic antidotes comprised of polymeric matrix including functional binders for melittin, aligned in a sense that they form a mold for a specific target, is a very efficient way to inhibit melittin [123]. Such NPs can be prepared by screening a library of NPs composed of various ratios of monomers containing functional groups complementary to the target peptide to select one with the highest intrinsic affinity [76,125]. Compared with biologic materials such as antibodies, synthetic materials offer an advantage because of their robustness and inexpensive production [126]. Limitations regarding nanoparticle utilization in therapeutic or imaging purposes are their potential toxicity [127], adsorption of serum proteins to NPs [128] that can alter or suppress their function, which leads to opsonization, followed by clearance from the bloodstream [129]. Many strategies were developed to prolong their blood circulation time and enhance tissue-specific uptake, mostly focusing on composition, size, surface charge, PEGylation and targeting functionality of NPs [130,131,132], but those can only be used to a limited extent in order to retain their binding characteristics [125].

### 2.3. Neutralizing Antibodies

Polyclonal antibodies (pAbs) have been utilized before for anti-PFP action, for example in passive protection of guinea pigs against anthrax infection with guinea pig pAbs [133]. In contrast to pAbs, monoclonal antibodies (mAbs) provide a continuous supply of homogeneous, well-characterized antibodies and represent an exquisite tool for specific binding. The traditional way of utilizing mAbs against toxins is by direct inhibition of their function [66], with several possible ways of preventing pore formation: Blockage of the toxin binding to its receptor, interference with oligomer assembly, or, in cases of PFPs with additional catalytic domains, binding to catalytic subunits of the toxin [133].

PFP-neutralizing antibodies were developed for pathogenic PFPs to complement the antibiotic therapy of various diseases, caused by those toxins. The first mAbs with PFP-neutralizing activity were developed in 1960s and inhibited pore formation of a CDC member streptolysin O [134,135,136,137]. In the 1990s, several mAbs were developed that neutralized the pore-forming activity of listeriolysin O and other CDCs, either by prevention of membrane binding, or preventing subsequent stages in the pore-forming process [137,138]. Jacobs et al. found a mAb that successfully bound to an undecapeptide, a peptide sequence conserved in all CDCs mediating the attachment of CDC monomers to the membrane, thereby neutralizing pore-forming activity of all tested toxins from the CDC family [139]. mAbs represent an excellent research tool. For example, it was possible to assess various conformational states of human perforin by using several mAbs [140].

Comprehensive reviews of anthrax-neutralizing mAbs have been published [45,141]. Anthrax toxin inhibitory mAbs can inhibit either receptor recognition by PA_83_ [142,143,144,145], proteolytic cleavage of PA_83_ [146], oligomerization step of the PA_63_ [147,148], or LF interaction with oligomerized PA_63_ [142,143,144,145,146,147,148]. A cooperative effect between two mAbs, one directed against LF and another against PA [146], or mAbs that exhibit synergistic protection when combined with established antibiotics [149,150], may prevent antibody-dependent enhancement of pathogenicity.

In addition to the anthrax toxin, many studies have been done on finding neutralizing mAbs against other pathogenic PFPs, such as toxin B from *Clostridium difficile* [151], *S. aureus* α-toxin [152,153], *Clostridium perfringens* ε-toxin [154,155], and others. Interestingly, some broad-spectrum mAbs were found to inhibit disparate PFPs, for example α-toxin together with four different types of leukotoxins [156]. In the case of human perforin, it was shown that commercially available monoclonal antibody Pf-80 (Mabtech) could inhibit its permeabilizing activity without affecting its binding to membranes [140,157].

Drawbacks of mAbs encompass their difficult production, accessibility of suitable hybridoma cell line, instability, and low yield of some hybridomas, side-effects, low tissue penetration, and high production cost [158,159,160]. However, those limitations are very well handled nowadays with utilization of different expression systems, and alternative selection techniques, such as phage display, and production of smaller antibody mimetics with retained or even improved antibody characteristics [158,161,162].

### 2.4. Antibody-Derived Scaffolds and Antibody Mimetics

Due to their high specificity and binding affinity, antibodies are still the most abundantly used proteins in various diagnostic assays and other molecular recognition purposes [163]. Bivalency, completely human origin, and long plasma half-life are undoubtedly the very desired advantages of IgG molecules, but on the other hand they are also relatively large and unstable, composed of multiple domains, and need disulfide formation and glycosylation for their activity, which makes their production laborious and costly [164]. These drawbacks, together with intellectual property rights that are bound to the majority of antibodies present on the market, motivate the development of alternative scaffolds which present an increasing role in biotechnology and medicine [164,165,166].

Most frequently used antibody fragments and non-immunoglobulin binders are antigen-binding fragments (Fabs and F(ab’)_2_s), single chain variable fragments (scFvs) (Figure 3d), variable fragments of heavy chain antibodies (V_H_Hs, also known as nanobodies), variable domains of sharks’ immunoglobulin new antigen receptors (V_NAR_), and adnectins (10th fibronectin type III domain derivatives, called also monobodies). They are easy to produce as recombinant proteins by bacterial cells in a fully functional form due to their small size, absence of disulfides, and no need for post-translational modifications [167]. Their advantages are also high solubility, excellent thermal stability, and allowance of complex sequence variation [168]. They are selected mostly by directed evolution of naïve libraries of a chosen scaffold, where the consensus areas important for correct folding are preserved, and the predicted epitope is subjected to diversification. This is performed by panning against the desired target with one of the display techniques, most commonly phage, cell-surface, mRNA, or ribosome display [169].

There are several examples of antibody fragment Fabs inhibiting different toxic PFPs. For example, Wild et al., 2003, selected a Fab that bound to a conformational epitope formed by PA_63_, inhibited LF interaction with PA_63_, and neutralized toxin substoichiometrically [170]. Moreover, a human/murine chimeric Fab was developed against LF [171] as well as against PA_83_ [172] of *B. anthracis*, all showing therapeutic potential for treatment of anthrax. Among others, scFvs directed against LF [173] and Cry1Ab toxin from *Bacillus thuringiensis* [174] were also developed. There are reports of isolated V_H_Hs and IgNARs against cholera toxin [175,176], *C. difficile* toxins [177,178], and others. Efficient neutralization of toxic PFPs can be achieved by using fusion proteins or bispecific binders that bind two subunits of PFP simultaneously and in such a manner inhibit the formation of pores [142]. Yang et al., 2015, produced a bispecific neutralizing construct, consisting of a mAb against epidermal growth factor receptor (EGFR) and a member of CDC, perfringolysin O (PFO), reversibly inhibited by an adnectin. The mAb enabled targeted delivery, whereas the adnectin ensured inactivity of PFO in the extracellular environment. After endocytosis, the adnectin dissociated from PFO, which lead to pore formation on membranes of endocytic compartments and the release of co-targeted protein with therapeutic effect [179]. Protein fusions can also aim for improved stability, such as scFv fused to the human antibody light chain constant κ domain (fusion called scAb), that bound to the PA_83_ of the anthrax toxin and as such acted as a competitor of the cellular receptor for PA_83_ binding [133]. It protected against anthrax toxin challenge in vitro and in vivo and was stable at elevated temperatures and highly resistant to deactivation in serum.

### 2.5. Polyvalent Inhibitors

Polyvalency refers to simultaneous binding of multiple ligands provided by one entity (inhibitor) to complementary receptors on the other (PFPs) [180]. Such polyvalent interactions are much stronger than the corresponding monovalent interactions and the principle has been successfully applied to block PFP activity with synthetic polymeric molecules, so called polyvalent inhibitors (PVIs).

Adequate matching when designing PVIs for pore formations can be achieved with a symmetric rigid cyclic scaffold (Figure 3e), one of the most studied being cyclodextrins [181]. Based on the number of sugar units in a molecule, cyclodextrins are divided into three groups, namely α, β, and γ. β-cyclodextrins are seven-fold symmetrical cyclic molecules with a hydrophobic cavity that mimic the symmetry of heptameric pores. They partially block pores of staphylococcal toxin α-toxin [20]. Their blocking efficiency can be further enhanced by addition of positively charged groups [182,183] or methylation and combination with cholesterol [184]. PA_63_ and LF of anthrax toxin were inhibited by β- as well as eight-fold γ-cyclodextrin derivatives [21,185,186,187,188], whereas six-fold α-cyclodextrins were ineffective. Additionally, β-cyclodextrins effectively inhibited C2 toxin of *Clostridium botulinum*, ι-toxin of *C. perfringens* and CDT binary toxin of *C. difficile* [189,190,191]. Mourez et al., 2001, identified a 12-amino acid residue peptide that weakly bound to the heptameric PA_63_ but not to monomeric PA. The peptide was synthesized chemically and shown to inhibit the interaction between PA_63_ and its ligands, EF and LF, albeit weakly. To generate a more potent form, they produced a PVI consisting of multiple copies of the synthetic peptide covalently linked to a flexible polyacrylamide backbone. The resulting construct was 7000-fold more potent than the monomeric form, owing to its ability to form multiple links to the oligomeric target [192]. The potency of inhibition can be further increased by a combination of the approaches mentioned above. For example, seven copies of an inhibitory peptide against heptameric PA_63_ were attached to a β-cyclodextrin scaffold, thereby producing a heptavalent inhibitor. This resulted in more than a 10^5^-fold increase in inhibition in comparison to the monomeric peptide [22].

Furthermore, prepores can be neutralized by peptide-functionalized liposomes. In the case of anthrax (PA_63_ heptamer) inhibitors, the liposomes measured approximately 50 nm in diameter and contained specific prepore-binding peptides fused to phospholipid headgroups [193]. The inhibitory effect was achieved at very low concentrations, they were active in vivo [191] and their further development has been made easier due to several FDA-approved liposome formulations as adjuvants [194] and drug deliverers [195,196]. Other forms of PVIs are dendrimers with functionalized ligands [197,198], rigid scaffolds fused with carbohydrate (instead of peptide) ligands [199], and receptor-directed PVIs [200] that diminish the consequences of toxic PFPs’ action by blocking host proteins. PVIs of PFPs have been recently reviewed by Yamini and Nestorovich, 2016 [201].

### 2.6. Receptor-Like Decoys for Pore-Forming Toxins

Another approach with a clinical potential for inhibition of various different types of PFPs was proposed by Bradley et al., 2001 [42], and further developed by several research groups [84,85,202,203,204,205]. The idea is to capture proteins that physiologically bind to receptors or cellular membranes with a surrogate system that mimics the natural one and consequently make the PFP unavailable for binding to its biological receptor in vivo [84,206]. This approach offers significant advantages compared to conventional strategies relying primarily on structure-specific epitope binding (e.g., antibodies) where we are usually faced with a high cost of antibodies and dosage requirements, the highly malleable nature of some of the PFPs, which implies they can easily mutate to become resistant to antibody-binding [73,207], and a serious challenge to devise an effective detoxification platform against bacterial infections of a very diverse range of toxic PFPs [208,209]. The superiority of receptor-like decoys over antibody-based antitoxins lies in their ability to accurately mimic the natural receptor while being less sensitive to natural or artificial changes in the PFPs’ primary structure [210,211].

There are numerous examples of surrogate receptors or membrane mimics that act as decoys to preoccupy and neutralize toxin actions. Soluble forms of extracellular domains of both anthrax receptors (TEM8 and CMG2) function as potent antitoxins that can protect cultured cells from intoxication, presumably by acting as receptor decoys to prevent PA_83_ from binding to cell-surface receptors [42,43,86,207,210]. There are various improved versions of receptor-like decoys. Wycoff et al., 2011, for example, fused the extracellular domain of CMG2 with a human Fc (fragment crystallizable) of IgG. The inclusion of the Fc domain allows efficient purification with protein A, causes dimerization (which increases size), and induces recycling and retention by interaction with the neonatal Fc receptor. The fusion protein efficiently neutralized PA_83_ as well as mutant forms of PA_83_ that were not successfully recognized by anti-PA monoclonal antibodies in vitro and in vivo [211].

An ingenious rationale that serum lipoproteins and membrane lipid extracts dispersed in water bind to and inhibit lethal and cytolytic activity of PFPs [212,213] was further developed by Hu et al., who named their receptor-like decoys “nanosponges” (Figure 3f). Consisting of erythrocyte membrane-coated nanoparticle system, they nonspecifically absorbed a broad spectrum of PFPs, thereby targeting the universal membrane-binding mechanism and offering an all-purpose PFP decoy strategy to absorb various types of PFPs regardless of their molecular structures [84,85]. Nanosponges have been so far effective in vivo at neutralizing α-toxin (also MRSA strain infections), streptolysin-O, and melittin [84,205,209], however a similar approach would probably be effective for all PFPs. Instead of an erythrocyte membrane, Henry et al. utilized artificial liposomes, containing higher than in vivo concentrations of cholesterol and sphingomyelin, resulting in inhibition of several staphylococcal and streptococcal toxins [206]. A similar approach can be practiced to capture PFPs that bind to specific receptors rather than to the membrane itself. Polyzos et al., 2007, for example, entrapped gangliosides G_M1_ into surfactant mesophase and the construct functioned as a polyvalent inhibitor of cholera toxin [214].

### 2.7. Dominant Negative Mutants

Dominant negative (DN) mutants (Figure 3g) are defective proteins that retain interaction capabilities but are inactive and possess the ability to inhibit the phenotype of the wild-type protein when mixed together [87]. For example, the DN mutant of the monomeric unit of VacA that is involved in oligomerization in the pore-forming process was able to block the pore-forming activity. The mutant PFP lacked the amino-terminal hydrophobic segment and did not exhibit any detectable defects in secretion, binding to membranes, oligomerization, or uptake by cells, but it failed to induce the vacuolization of the toxin, and was consequently non-cytotoxic. When combined with the wild-type toxin, the dysfunctional mixed oligomers comprised of both mutant and wild-type VacA monomeric components were formed and the cytotoxic activity of the latter was inhibited [215].

Inhibition of the anthrax toxin PA component pore formation by DN mutants was reported independently by two groups. Sellman, Mourez, and Collier, 2001, identified a PA with double mutation with properties of a DN mutant. It co-assembled with the wild-type PA and generated defective prepores impaired in pore formation and in translocating EF and LF across the endosomal membrane. Despite these malfunctions, the proteolytic activation of PA and self-assembly of the toxin remained unaffected [216]. Singh et al., 2001, found another mutated PA with similar effects. They showed that a mixture of DN mutant PA and wild-type PA completely inhibited toxin activity in vitro and in vivo [217]. Later, a whole map of possible sites where a single amino acid replacement on PA_63_ can give a DN phenotype was proposed [218]. They found that DN mutations are only feasible in pore-forming domain II of PA and specifically affect pore formation and translocation. Furthermore, none of the DN mutations tested significantly impaired the immunogenic properties of PA. Hence, DN forms of PA may have the potential to serve both as direct inhibitors of toxin action and as inducers of protective antibodies. These properties may make them useful for post-exposure therapy and prophylaxis against anthrax [41,48]. As antibiotic treatment cannot provide full protection against relapse or subsequent exposure to anthrax, some claim conjunctive antibiotic treatment and vaccination with DN inhibitors would be an ideal option [219]. There are numerous other examples of DN mutants inhibiting pore formation by anthrax [220,221] and other pore-forming toxins, including *C. perfringens* ε-toxin [83], *B. thuringiensis* Cry1Ab [222], and *E. coli* cytolysin A [223]. These examples imply that toxins acting through oligomeric complexes are amenable to dominant-negative inhibition, a paradigm that could be broadly applied.

Notably, many of toxin inhibitors devised so far are either derivatives of a toxin component or receptor. In addition to DN mutants and receptor decoys, toxin derivatives can act as competitive inhibitors for receptor binding. For example, PA with a mutated furin site competes with the wild-type protein for receptors, and the mutation in the N-terminal domain of LF competitively inhibits binding of EF and LF to PA_63_ [41]. However, DN forms of toxins are far more potent toxin inhibitors than those that merely compete for receptor binding, and for this reason the competitive inhibition of receptor binding is not widely used for toxin neutralization.

## 3. Conclusions

The biological activity of toxic PFPs, which represent the largest group of bacterial cytotoxic proteins, can be blocked by various strategies and inhibitor designs. Here we describe general approaches by which the pore-formation step can be inhibited together with a number of studies in which these approaches have been employed. All presented strategies possess the ability to potently inhibit PFPs. The suitability of the strategy hence depends mostly on the aim of application.

Specific inhibitors of pore-formation have been primarily developed to affect the course of disease and its symptoms. As such, drugs targeting toxic PFPs could help limit the extent of infection, aid in preventing systemic spreading when a localized infection is present, and prevent toxic PFP-mediated tissue destruction (e.g., in *S. pneumoniae* or *S. aureus* pneumonia or clostridial myonecrosis). Such drugs could also be used to prevent problematic nosocomial infections (e.g., preventive administration during surgery or the use of catheters) [38] or as an adjunct to antibiotic therapy—for co-administration with existing antibiotics for delaying the infection and therefore providing time for antibacterial agents or the immune system to clear an infection [86,170,192,200,207]. Probably, a cocktail of peptides or protein fragments that interfere with several of the protein–protein interactions required for toxin action would be the most efficacious therapy [224].

Besides utilization of inhibitors that prevent specific steps in pore formation of PFPs in therapeutic applications, these inhibitors are also a valuable tool in studies of the mechanism of action of PFPs [137,140,217], protein expression in vitro and in vivo [157,225,226], determination of structure–function relationship [81,218,227,228], detection of cytotoxic cells that express PFPs [225], testing PFPs’ activity with functional assays [157], probing the distribution, orientation, and mobility of membrane receptors [229], etc. PFPs also play an important role in a wide range of recent advances in nanotechnology [230,231]. By gaining new findings about their pore-forming mechanisms, we could use them more effectively in developing technologies, such as molecular sensing and detection [232,233,234,235], DNA sequencing [236,237,238], monitoring of chemical and biochemical reactions, development of biocompatible nanotransistors [239], and novel drug delivery systems and targeted killing of cells [179,230,240], as well as develop new applications. As the technology proceeds, the potential space for applications of PFP inhibitors will also grow.
